# Radiological features of primitive neuroectodermal tumors in intra-abdominal and retroperitoneal regions: A series of 18 cases

**DOI:** 10.1371/journal.pone.0173536

**Published:** 2017-03-20

**Authors:** Xiaoping Yi, Wenguang Liu, Youming Zhang, Desheng Xiao, Hongling Yin, Xueying Long, Li Li, Hongyan Zai, Minfeng Chen, Wenzheng Li, Lunquan Sun

**Affiliations:** 1 Department of Radiology, Xiangya Hospital, Central South University; Changsha, P.R. China; 2 Postdoctoral Research Workstation of Pathology and Pathophysiology, Basic Medical Sciences, Xiangya Hospital, Central South University; Changsha, P.R. China; 3 Department of Pathology, Xiangya hospital, Central South University; Changsha, P.R. China; 4 Imaging Diagnosis and Interventional Center, Sun Yat-sen University Cancer Center; Changsha, P.R. China; 5 Departments of General Surgery, Xiangya Hospital, Central South University; Changsha, P.R. China; 6 Departments of Urology, Xiangya Hospital, Central South University; Changsha, P.R. China; 7 Center for Molecular Medicine, Xiangya Hospital, Central South University; Changsha, P.R. China; Universidad de Navarra, SPAIN

## Abstract

**Objectives:**

To characterize the imaging and clinicopathological features of primitive neuroectodermal tumors (PNETs) arising in intra-abdominal and retroperitoneal regions.

**Methods:**

Eighteen patients with histopathologically proven intra-abdominal and retroperitoneal PNET were enrolled; computed tomography was performed for all cases, and magnetic resonance imaging was performed for a single case. Typical computed tomography and magnetic resonance imaging findings, including morphology, texture and enhancement features, as well as clinicopathological characteristics and prognosis data were retrospectively analyzed.

**Results:**

Of eighteen PNET patients, fifteen were male and three were female, with a median age of 36 years (range, 2–65 years). The onset of symptoms was most often nonspecific and insidious. The mean tumor diameter was 7.2 cm (range, 3.0–12.1 cm), with necrosis in fifteen cases, cystic changes in eight, partition structure in five, calcification in five, hemorrhage in two, and mural nodules in one. Contrast enhanced computed tomography showed multiple tiny feeding arteries within the masses in six cases, resulting in a crab-like appearance, and mild ring enhancement pattern in five cases. Eleven cases showed surrounding invasion and metastasis. Of the eighteen PNET cases, nine cases showed smooth, well-defined margins, and nine cases had irregular, ill-defined margins. A median survival was 10.0±1.6 months. However, chemotherapy had efficacy on patients even those with advanced disease.

**Conclusions:**

Primary intra-abdominal and retroperitoneal PNETs are rare, and imaging features documented here may help the diagnosis of this severe disease. Notably, two signs present in retroperitoneal PNET tumors, including a mild ring enhancement pattern and a crab-like appearance of the tiny feeding arteries, may have the potential to help us improve the ability to make a relatively reliable diagnosis.

## Introduction

Primitive neuroectodermal tumors (PNETs) are a group of extremely rare malignant tumors that originate from the neuroectoderm and are composed of primitive undifferentiated small round cells [1,2]. Based upon the 2013 WHO classification of tumors of soft tissue and bone, PNETs belong to the Ewing’s sarcoma family of tumors, and more than 85% of PNET and Ewing sarcoma tumor cells exhibit the same chromosomal translocation (t(11;22)(q24;q12)) [3,4]. Despite the differences in their degree of neural differentiation, these tumors share similar characteristics, such as clinical appearance, pathological features, cellular molecular and genetic characteristics and prognoses. Generally, PNETs can be further classified into two types: central PNET (cPNET) and peripheral PNET (pPNET) [1]. pPNETs arise outside the central and sympathetic nervous system, and their overall incidence accounts for 1% of all sarcomas [5]. pPNETs are commonly located in the soft tissues of the thoracopulmonary region (Askin tumor), paraspinal region, and limbs. Tumors originating in the intra-abdominal and retroperitoneal regions, head and neck regions and bone are relatively rare [2,6–8].

Although some studies involving intra-abdominal and retroperitoneal PNETs have been reported and some radiological features of these tumors have been described, this disease is still poorly recognized and easily misdiagnosed due to the lack of a typical clinical presentation, a low incidence in the clinic and an insufficient number of radiological studies [1,2,6,9–11]. Thus, making a relatively precise diagnosis of intra-abdominal and retroperitoneal PNETs before surgery is still challenging for radiologist or clinicians. Awareness of the characteristic imaging of this disease and its underlying pathological basis has yet to be achieved.

Herein, we conducted a retrospective study of 18 patients with pathologically proven intra-abdominal and retroperitoneal PNETs at our hospital to identify computed tomography (CT) and magnetic resonance imaging (MRI) characteristics of this rare disease. The treatment and prognosis of this disease are also discussed.

## Materials and methods

### Research design

**Fig 1:**
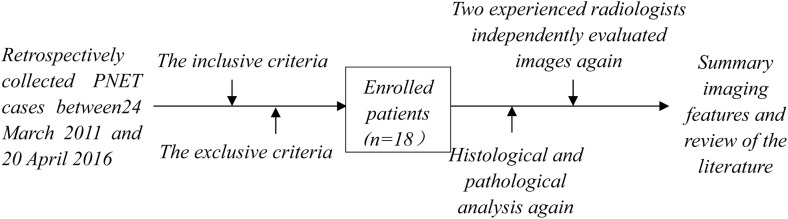
Flow Chart of the Present Study.

The research design of this retrospective study is shown in **Fig 1**.

### Patient selection

Between March 24, 2011 and April 20, 2016, 70 consecutive patients with pathologically confirmed pPNETs (extraosseous primary tumors) were admitted to our hospital. The participant information was de-identified and obtained from our hospital database.

The inclusion criteria were patients with PNETs originating in the intra-abdominal or retroperitoneal region. The exclusion criteria were patients without CT or MRI examinations before surgery or incomplete clinical data (medical records, pathological data, follow-up data, etc.).

### Radiological examination

CT scans were obtained using a spiral CT scanner (Toshiba 320 CT, Acquilion One, Japan and Siemens Dual-source CT, Somatom Definition, Germany). The scanning parameters were as follows: for Acquilion One—120 kVp, 200 mA, 1-mm section thickness and 5-mm reconstruction thickness; for Somatom Definition—120 kVp, 223 mA, 1-mm section thickness and 5-mm reconstruction thickness. All patients underwent unenhanced and enhanced scans. Iohexol, a nonionic iodine contrast agent, was used in enhanced scans at a dose of 1.5 ml/kg (iodine concentration of 300 mg/ml) and an injection flow rate of 3–4 ml/s. Images were obtained at each phase, which included plain scan, arterial phase (30 s), venous phase (60–70 s) and delayed scan (180 s).

One patient was examined with a 1.5 T MRI unit (Siemens, Sonata, Germany) using an eight-channel array coil. The scanning parameters were as follows: 4-mm section thickness, 4-mm intersection gap, 30×30 field of view and 384×269 matrix. A respiration triggered turbo spin-echo (TSE) T1-weighted sequence [TSET1 with a 500-ms repetition time (TR), an 11-ms echo time (TE)] and a TSE T2-weighted, fat-suppressed (TSET2) sequence (3000 ms TR, 97 ms TE) were used. TSET1-enhanced MRI was performed using Gd-DTPA as the contrast agent. A dose of 0.2 mg/kg was used with an injection flow rate of 1.5 ml/s. Arterial phase imaging was performed approximately 25–30 s after injection, venous phase imaging approximately 50 s after injection and delayed phase imaging approximately 5 min after injection.

### Imaging, pathological and prognosis analyses

CT and MRI images were reviewed independently by two experienced abdominal imaging radiologists (with more than 10 years of experience). The findings were recorded by consensus. CT and MRI characteristics were investigated, with emphasis on the location, size and margin of the lesion (well-defined or poorly defined); internal content (homogeneous or heterogeneous, density/intensity, with or without necrosis, hemorrhage, cystic degeneration, septation or calcification); pattern of enhancement; and changes of the adjacent structures. The lesion size was measured at its greatest diameter. For the internal content, the “cystic” lesions were defined as those containing typical fluid-like attenuation or signal intensity on CT or MRI and showing rim-like enhancement on contrast-enhanced CT and MRI. Density on non-contrast CT scans and signal intensity on T1- and T2-weighted MRI images were also documented subjectively, as were enhancement patterns on contrast-enhanced CT and MRI. The pattern of enhancement was categorized as homogeneous or heterogeneous. In addition, we evaluated the blood supply arteries and surrounding tiny feeding arteries of the tumor. The mild ring enhancement of the tumor was observed in the venous phase.

The pathological sections (including the hematoxylin and eosin (HE) and immunohistochemical (IHC) staining) of all enrolled PNET patients were reanalyzed independently by two experienced pathologists. The findings were recorded by consensus. The expression of cluster of differentiation 99 (CD99), neuron-specific enolase (NSE), vimentin (VIM), synaptophysin and S100-protein was examined.

Statistical analysis of survival was performed using the Kaplan-Meier method, and the results were examined using the log-rank test. A P value less than 0.05 was considered statistically significant. Statistics were calculated using SPSS 15.0 (SPSS Inc., Chicago, IL, USA).

### Compliance with ethical standards

#### Ethical standards

This study was conducted in accordance with the Declaration of Helsinki, all its amendments and national regulations. This retrospective study was approved by the hospital’s Institutional Review Board (No. 201512538). Written or verbal informed consent was obtained from all patients or their immediate families.

## Results

### Clinicopathological data

According to the study design, 18 consecutive patients with pathologically proven PNET arising in intra-abdominal and retroperitoneal regions were enrolled in this study. The clinicopathological data are summarized in **Table 1**. Detailed data can be found in **Table 2**.

**Table 1:** Clinical Data and Main Radiologic Findings in 18 Patients with pPNETs.

	All	Intra-abdominal	Retroperitoneal
Case	18	3	15
Age (years old)	36(range, 2–65 years)	54 (range, 3–65 years)	36(range, 2–54 years)
Sex			
M	15	2	13
F	3	1	2
Location		Bladder (1); Ascending colon (1); Mesentery (1)	Adrenal gland region (5); Renal (3); Renal hilum (2); Hepatogastric gap (2); Presacral region (1); Head of the pancreas (1); Lesser curvature of stomach(1);
Size	7.2	7.1	7.2
Margin			
Well-defined		1(33.3%)	8(53.3%)
Ill-defined		2(66.7%)	7(46.7%)
Necrosis		2(66.7%)	13(86.7%)
Hemorrhage		0	2(13.3%)
Cyst		1(33.3%)	7(46.7%)
Septa		0	5(33.3%)
Calcification		1(33.3%)	4(26.7%)
Enhancement			
Homogenous		1(33.3%)	3(20.0%)
Heterogeneous		2(66.7%)	12(80.0%)
Tiny feeding arteries		1(33.3%)	6(40.0%)
Mild ring enhancement		0	5(33.3%)

Note: M, male; F, female.

**Table 2:** Clinical data.

Cases no.	Age (years)	Sex	Clinical Presentation	Location	Treatment	Outcome (month after first diagnosis)	Metastasis
1	54	M	Hematuria, urinary frequency	bladder wall	TR	Dead (17)	None
2	12	M	Waist and abdominal pain	Right adrenal gland	PR+chemo	Dead (8)	Around the renal hilum lymph node metastasis
3	5	F	Abdominal distension, urination and defecation difficult	Presacral area	PR	Dead (18)	Rectal posterior wall and levator ani muscle involvement
4	51	F	Upper abdominal pain	Hepatogastric gap	BI	Dead (3)	The greater omentum, retroperitoneal lymph node metastasis
5	27	M	Incidentally detected	Left adrenal gland	PR	Dead (5)	Retroperitoneal lymph node metastasis
6	36	M	Upper abdominal pain, jaundice	Head of the pancreas	BI	Dead (2)	None
7	48	M	Incidentally detected	Right adrenal gland	PR+chemo	Dead (10)	None
8	36	M	Abdominal pain	left renal hilum	BI+chemo	Alive (24)	Left kidney, left adrenal gland, left renal artery and vein, abdominal cavity and abdominal aorta involvement
9	40	M	Left waist pain	Left renal calices	PR	Dead (12)	None
10	2	M	Incidentally detected	Left adrenal gland	BI +chemo	Dead (15)	None
11	42	M	Upper abdominal and xiphoid pain	Lesser curvature of stomach beside	PR	Dead (10)	None
12	65	M	Lower abdominal pain	Ascending colon	BI	Dead (4)	Liver, mesentery, retroperitoneal lymph node metastasis
13	22	M	Left waist hold back inflation	Left adrenal gland	TR	Dead (11)	Retroperitoneal lymph node metastasis
14	3	F	Abdominal pain	Mesentery	BI+ chemo	Dead (5)	Retroperitoneal lymph node metastasis
15	44	M	Incidentally detected	Right renal	TR	Dead (11)	None
16	20	M	Lower left abdominal pain	Left renal	TR	Dead (16)	Left renal vein tumor thrombus, Left renal hilar lymph node metastasis
17	16	M	Left abdominal pain, hematuria	Left renal	TR + chemo	Alive (17)	Left renal vein tumor thrombus
18	37	M	Abdominal pain	Hepatogastric gap	BI	Dead (2)	Invasion into the right pleural

**Note:** M, male; F, female; TR, radical resection; PR, partial resection; chemo, chemotherapy; BI, biopsy.

The study was composed of eighteen patients (fifteen males and three females) with a median age of 36 years (range, 2–65 years old). The onset of symptoms was most often nonspecific and insidious, including mild low back pain or abdominal pain in eleven patients, hematuria and polyuria in two, and abdominal distension accompanied by bladder bowel dysfunction in one. The tumors in the remaining four patients were incidentally detected.

### CT and MRI findings

CT (n = 18) and MRI (n = 1) images obtained from these eighteen patients were retrospectively reviewed. Among them, fifteen had a mass located in the retroperitoneal space and three in the intra-abdominal region (Table 1**)**. Detailed CT and MRI findings are shown in **S1 Table**.

**Fig 2:**
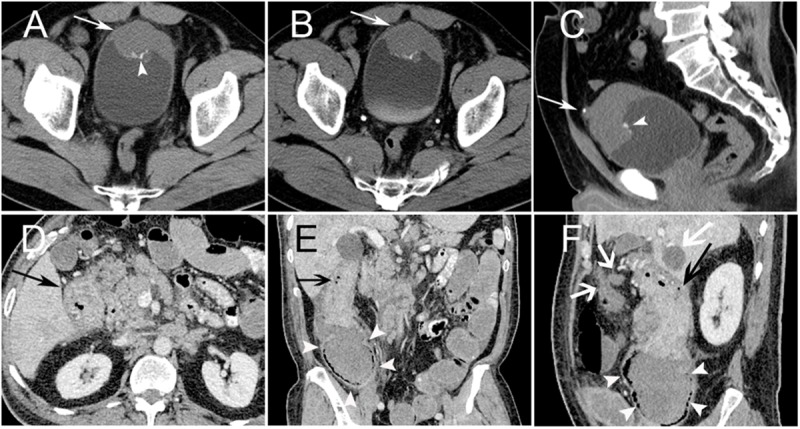
PNET Arising in the Intra-abdominal Region. PNET of the bladder in a 54-year-old male (Case no. 1). Non-contrast CT image showed the anterior bladder wall with uneven thickening (arrow) and scattered punctuate calcification (arrowhead) (A). Contrast-enhanced CT showed mild homogenous enhancement of the mass (arrow) (B). Sagittal CT image showed calcification located on the mass surface (arrowhead), and a urachal stone was demonstrated (arrow) (C). Ascending colon PNET in a 65-year-old male (Case no. 12). CT images showed a locoregional ascending colon wall with uneven thickening and intermediate heterogeneous enhancement (black arrow) (D). Ascending colon locoregional luminal stenosis (arrow) (E, F) showed an incomplete intestinal obstruction with proximal colon dilatation (white arrowheads) (E). Retroperitoneal lymph node involvement and liver metastases were observed (white arrow) (F).

Tumors arising in the intra-abdominal region derived from intra-abdominal organs; one mass in the bladder wall (Fig 2A–2C), one in the ascending colon (Fig 2D–2F), and one in the mesentery. The maximum diameter of the masses ranged from 4.0 to 10.8 cm, with a mean diameter of 7.1 cm. Of the three cases, two had an ill-defined margin, and one was well defined. Unenhanced CT revealed a mild-to-moderate heterogeneous iso-dense mass in all three cases. On contrast-enhanced imaging, all three masses showed intermediate enhancement. In addition, scattered fine-and-punctate calcifications were found on the edge of the bladder mass. Retroperitoneal lymph node involvement and multiple liver metastases were observed in the ascending colon case (**Fig 2F**). Multiple retroperitoneal metastatic lymph nodes (mainly around the iliac vessels) were observed in the mesentery case.

**Fig 3:**
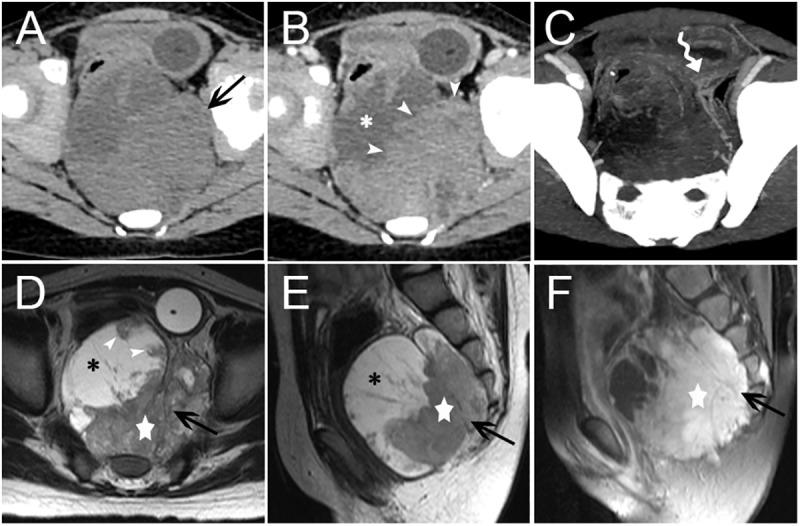
Presacral Region pPNET in a 5-year-old Female (Case no. 3). CT images showed an irregular, mixed solid cystic mass (arrow) with an indistinct margin in the presacral region (A). The necrotic or cystic portion (asterisk) did not show clear enhancement, but the solid portion (arrowheads) presented with intermediate heterogeneous enhancement (B). Blood supply arteries could be observed (curved tail arrow) (C). Non-contrast MRI images showed the solid components (five-pointed star) of the mass (arrow) was isointense on T1WI images and mildly, heterogeneous hyper-intense on T2WI images. The lateral necrotic or cystic portion (asterisk) showed hypo-intensity on T1WI images and heterogeneous hyper-intensity on T2WI images. Mural nodules were observed (arrowhead) (D-F). On contrast-enhanced MRI images, the solid components (five-pointed star) of the mass showed marked heterogeneous enhancement, with low signal separation lines within the tumor (F).

**Fig 4:**
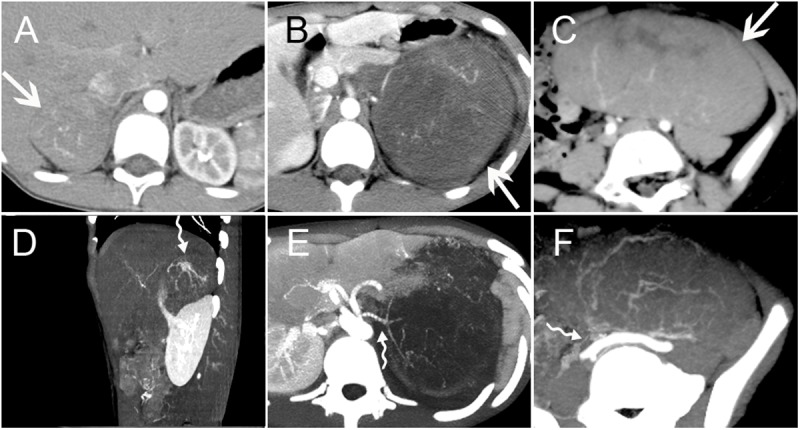
Tumor Blood Supply. Three PNET cases with masses arising in the right adrenal gland region (A/D) (Case no. 2), left kidney (B/E) (Case no. 17) and mesentery (C/F) (Case no. 14) (arrows). Contrast-enhanced arterial phase CT images showed circuitous lines of small blood vessels within the masses. Tiny feeding arteries could be observed inside the mass, showing a crab-like appearance (curved tail arrow) (D-E).

**Fig 5:**
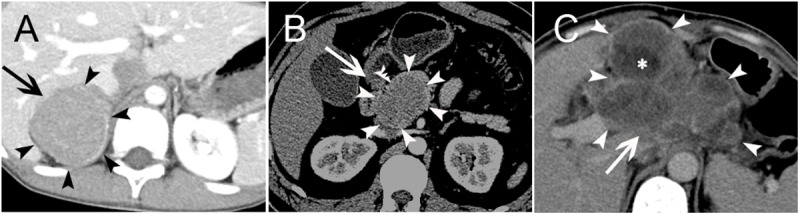
Mild Ring Enhancement. Three PNET cases with masses arising in the right adrenal gland region (A) (Case no. 2), pancreatic head (B) (Case no. 7) and hepatogastric space (C) (Case no. 4) (arrows). Contrast-enhanced CT images showed a mild ring enhancement on portal venous phase (arrowheads), which was more than a half circle surrounding the mass (A-C). The lesions all showed intermediate heterogeneous enhancement. Several patchy cystic areas or septation areas without enhancement (asterisk) could be found in the lesions (C).

Of the fifteen retroperitoneal PNET cases included in this study, fourteen single solid masses and one single mixed solid cystic mass were detected. Among them, five were located in the adrenal region bilaterally, three in the kidney bilaterally, two in the renal hilum, two in the hepatogastric space, one in the presacral region, one in the head of the pancreas, and one in the lesser curvature of the stomach. The size of these masses ranged from 3.0 to 12.1 cm, with a mean diameter of 7.2 cm. Eight cases had a well-defined margin, and the other seven cases were ill-defined. On non-contrast CT scan (n = 15), the attenuation of all masses was mild-to-moderate, heterogeneous hypo-attenuated compared with the adjacent muscle. All fifteen patients underwent contrast-enhanced CT scan, and the masses exhibited mild or intermediate heterogeneous (n = 12) and homogeneous (n = 3) enhancement, whereas the cystic portion of the masses usually demonstrated a peripheral rim enhancement pattern. Necrosis was found in thirteen cases, partial or peritumoral cyst degeneration in seven, septa in five, calcification in four, hemorrhage in two, and mural nodules in one (**Fig 3**). Clearly defined blood supply arteries, which are usually located at the edge of the tumor and are relatively dense in some regions, were found in six cases. The arteries were usually located on the side of the tumor, which was close to the artery supplying the corresponding region where the mass was located. Interestingly, tiny feeding arteries extending inside the tumor from such regions could be observed in six cases, and they presented with a crab-like appearance (**Fig 4**). A ring-like enhancement pattern was found in five cases, which indicates that the enhanced ring occupied more than a half circle surrounding the mass on portal venous phase images (**Fig 5**). Among the fifteen cases, multiple perirenal drainage veins were observed in one case, and an enlarged tortuous tumor-draining vein that converged into the right external iliac vein was observed in another case. In general, the majority of tumors displaced the surrounding tissues. However, tumor invasion into adjacent tissues and organs (such as the left portal vein, inferior vena cava (IVC), celiac axis, renal pelvis, left renal vein and artery) was observed in four cases, and a left renal vein tumor thrombus was confirmed in three cases. Three cases with masses arising in the kidney showed increased volume of the kidney; however, they were well confined within the renal contour. A right pleural invasion was observed in one case.

### Treatment and follow-up

Surgical resection was performed for eleven patients, including radical resection in five patients (group one) and partial resection in six patients (group two). Biopsy was performed for seven patients (group three), four of which were performed during exploratory laparotomy. All patients were recommended to receive systemic chemotherapy; however, only six patients were eventually treated with chemotherapy, and the remaining twelve refused due to economic considerations. Detailed data are provided in **Table 2**.

**Fig 6:**
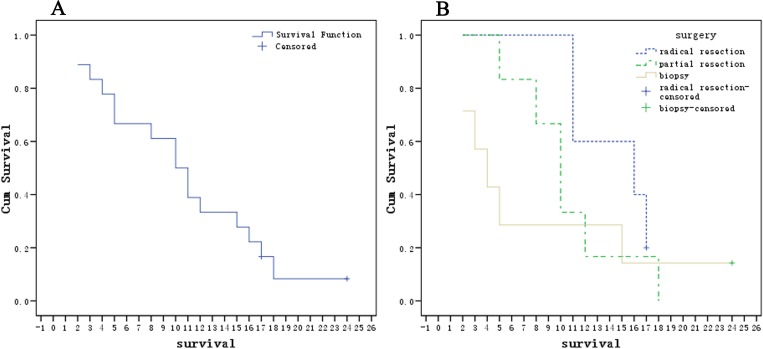
Kaplan-Meier Curves for Patients with PNET in the Intra-abdominal and Retroperitoneal Regions. All patients (n = 18) (A). The 18 patients were divided into three groups based on different surgical methods: radical resection (n = 5), partial resection (n = 6) or biopsy only (n = 7) (B). Multiple comparisons between the three groups showed no significant differences (*p*>0.10).

The prognosis of these eighteen patients was extremely poor, with a median survival of approximately 10.0±1.6 months (**Fig 6A**) (detailed data are provided in **Table 2**). Group one had a relatively better prognosis (**Fig 6B**). Only two patients were still alive at the end of this retrospective study, and they belonged to group one (Case no. 17, 17 months) and group three (Case no. 8, 24 months) (the chemotherapy strategy employed is presented in **S2 Table**, CT images of six times are shown in **S1 Fig**), both of whom received systemic chemotherapy. The remaining sixteen patients ultimately died within 1.5 years of diagnosis (range, 2–18 months). In addition, three patients of group three who underwent systemic chemotherapy appeared to have a relatively longer survival (24, 15 and 5 months).

### Gross pathology and immunohistochemistry findings

**Fig 7:**
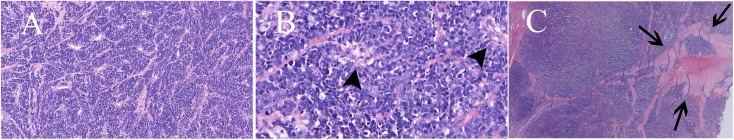
Pathological Findings for Patients with PNET in the Intra-abdominal and Retroperitoneal Regions. Two PNET cases with masses arising in the hepatogastric space (A-B) (Case no. 4) and the right adrenal gland region (C) (Case no. 2). HE staining showed that the tumor was composed of atypical small round cells with scant cytoplasm, which were arranged in nests with a small amount of stromal components (including collagen fibers and blood vessels) (A) (100×). Cells were arranged into nests and formed a ‘Homer-Wright rosette’ structure (B) (arrowhead) (400×). Stromal elements of the mass usually extended into the central region of the tumor, which showed a crab-like appearance (C) (arrow) (40×).

Most masses showed vague boundaries between the tumor edge and normal tissue, with significant necrosis, which were mostly consistent with the appearance on CT and MRI. Microscopically, these tumors were composed of atypical small round cells with scant cytoplasm, which were arranged in nests with a small amount of stromal components (including collagen fibers and blood vessels) (**Fig 7A**). Cells were arranged into nests or cords with rosettes (Homer-Wright rosettes) (**Fig 7B**). Generally, a significant amount of space in the tumor was occupied by dense tumor cells with scattered stroma. Interestingly, stromal elements of the mass usually extended into the central region of the tumor, which showed a crab-like appearance similar to that observed on the CT images (**Fig 7C**). IHC analysis of the eighteen tumors revealed that seventeen (94.4%) were positive for CD99, ten (55.6%) were positive for NSE, twelve (66.7%) were positive for vimentin and four (22.2%) were positive for S100 protein. Many cells demonstrated abnormal increased karyokinesis, with a Ki-67 index in the range of 20%-90%.

## Discussion

The incidence of PNETs arising in the peritoneal cavity and retroperitoneum accounts for approximately 14% of all peripheral PNETs [12]. Generally, patients with PNETs present with certain nonspecific symptoms, such as abdominal pain, back pain and hematuria [2]. Consistent with previous reports, our cases also usually manifested with insidious and nonspecific symptoms, including lower back or abdominal pain, abdominal distension, hematuria and polyuria. It is worth noting that four patients (22.2%) in our study were actually detected incidentally. Notably, incidental lesions that require timely treatment are increasingly being detected, which may be due to the development and wide-spread use of modern imaging modalities, especially in current China. Therefore, the radiologist’s role in differentiating lesions, such as PNETs, that do require surgical therapy from those lesions that do not is becoming increasingly important. Moreover, among the few cases that have been reported, neo-adjuvant chemotherapy may play an important role in the treatment of PNETs, especially in advanced patients [13–15]. Therefore, the preoperative non-invasive imaging diagnosis of PNET is even more important.

Generally, PNETs arising in the abdominal region tend to present as relatively large soft tissue masses with mean diameters greater than 5 cm [1,2]. On CT/MRI images, they usually appear as ill-defined, irregular or lobular moderate heterogeneous masses with cystic and necrotic areas, and rarely calcification and hemorrhage, as well as mild to moderate heterogeneous enhancement [2,6]. Our results are comparable to those of other studies; the maximum diameter of the masses in our cases ranged from 3.0 to 12.1 cm, with a mean diameter of 7.2 cm. The relatively larger size may have been due to the deep location of the mass, which often resulted in mild or even insidious symptoms of compression, even though these were not obvious until the tumor grew large enough.

Consistent with an earlier report [2], 53.3% (8/15) of tumors in this study had a well-defined margin. Notably, among the eight tumors, masses located in the adrenal region (n = 5) and renal calices (n = 1) had very clear boundaries. We hypothesize that this finding may be due to two reasons. First, the presence of abundant fat, which wrapped around the mass in the adrenal region, resulted in a sharp contrast and an obvious natural boundary between the two different tissues on CT/MRI images. Second, the rapid expansion of the tumor continuously compresses the surrounding connective tissues of the retroperitoneal space, resulting in a complete capsule. In the present study, we made the surprising observation that the proliferation activity (median Ki-67 index, 40%; range, 20%-90%) was relatively high in the 8 cases with well-defined margins. Given the rapid formation of the capsule, our hypothesis is that the capsule did not have enough time to be destroyed by tumor invasion despite the high malignancy and invasiveness of the tumor.

In our study, two tumors were located in abdominopelvic cavity organs, including the ascending colon and bladder, which are both hollow organs. According to previous reports, pPNETs arising in hollow organs (such as the colon and bladder) usually present as local wall thickening [16,17], as were the two cases in the present study. In addition, scattered punctuate calcification was found in the patient with bladder PNET, surrounding tissue invasion was observed in both cases, and distant metastasis was observed in the colon PNET patient. Notably, these nonspecific radiological signs may not be helpful when establishing a differential diagnosis because they may be common features of cancers arising in the bladder and colon. It is worth noting that bladder and colon PNETs presented with only mild to moderate enhancement patterns on enhanced images, which is completely distinct from that of bladder or colon cancer. Bladder or colon cancers tend to show clear heterogeneous enhancement after contrast agent administration.

Masses arose in bilateral kidneys of three renal PNET cases, resulting in an increased volume of the involved kidney. However, lesions did not cross the renal capsule, which indicates that they were well confined within the kidney contour. This finding is consistent with previous reports in the literature [5,6] and supports the notion that the tumor originated in the renal medulla, inducing invasive and expansive growth centered round the renal cortex.

A large mass with cystic degeneration and necrosis is a common feature of CT imaging for PNETs in the abdominopelvic cavity [2], where calcification is rare, occurring in less than 10% of cases [18]. In our cases, cystic and necrosis were indeed common. The thirteen retroperitoneal PNET cases with relatively larger diameters (mean size of 7.6 cm) all showed clear cystic degeneration and necrosis. In addition, the tumor blood supply arteries could be demonstrated in 86.7% (13/15) of cases. Moreover, tumor necrosis was usually observed in a region adjacent to the distal artery. Larger tumors are considered to be more prone to cyst formation and necrosis because localized ischemia and hypoxia are more common. Unfortunately, this imaging feature remains nonspecific and may not be helpful in the differential diagnosis of these tumors. Inconsistent with earlier studies, a higher rate of calcification was observed in this study, with 26.7% (4/15) of cases showing scattered punctate calcifications in the masses.

Based on our review of the literature, we identified two CT features in our fifteen retroperitoneal cases that have not been previously discussed seriously and may have the potential to become characteristic imaging features of retroperitoneal PNET. First, a mild ring enhancement sign was observed in 40.0% (6/15) of cases, including three cases located in both adrenal regions, two in the hepatogastric space and one in the pancreas. This CT feature may directly represent the enhanced tumor pseudo-capsule and may be a reflection of a high proliferative ability of the mass, as discussed above. Second, 40.0% (6/15) of cases clearly presented with tiny feeding arteries in the mass, which showed a crab-like appearance on enhanced images. This feature may partially reflect the distribution patterns of intra-tumoral stroma (see Results). In 53.3% (8/15) of cases, we did not observe obvious invasion of the adjacent vasculature. This finding somewhat resembles the appearance of lymphoma, another common type of ‘small round cell tumor.’ It is plausible that this phenomenon may be a common characteristic of small round cell tumors on imaging. Certainly, whether it is pertinent to the differential diagnosis of this tumor needs to be confirmed with additional cases.

The first-line treatment for pPNET is a multimodal treatment strategy consisting of surgery, neo-adjuvant or adjuvant chemotherapy or radiotherapy [18]. In particular, the effect of adjuvant chemotherapy on PNET has been evidenced. According to a previous study performed by Grier et al. [19] and Womer et al. [20], the 5-year event-free survival rate of PNET patients can be up to 65–73% after adjuvant chemotherapy. Therefore, it is not surprising that the patients’ 1-year survival rate was only 33.3% in our case, which is significantly lower than that in other reports, especially when considering the much lower proportion of patients (6/18, 33.3%) who received chemotherapy in our study. In addition, patients who underwent chemotherapy may also achieve a relatively better prognosis regardless of which surgery was performed and how advanced the disease was (as shown in the Results section of our study). Thus, systemic chemotherapy should be recommended to PNET patients to achieve relatively longer survival even for patients at an advanced clinical stage.

We acknowledge that the number of cases in this study was small, which may have affected the reliability of the conclusions. Due to the rarity of the disease, a small sample size is almost inevitable until a large multi-institutional randomized controlled trial (RCT) experiment can be performed. Therefore, further study with a large case number would be necessary to provide sound evidence for our results in the present study. Further limitations of the present study also include the lack of detection of translocation (EWS-FLI) in our patients at diagnosis, even though they were all evaluated with an extensive IHC panel.

## Conclusions

In summary, the imaging features of intra-peritoneal and retroperitoneal PNETs are nonspecific. They mainly occur in adults and usually present as large, ill-defined, irregular or lobular moderate heterogeneous masses with cystic and necrotic areas, and rarely calcification and hemorrhage, as well as mild to moderate heterogeneous enhancement. However, two signs present in retroperitoneal PNET tumors, including a mild ring enhancement pattern and a crab-like appearance of the tiny feeding arteries, may have the potential to help us improve the ability to make a relatively reliable diagnosis of this disease. The likelihood of PNET diagnosis should be considered when a diagnosis of a common tumor is not favored by imaging features.

## Supporting information

S1 Table Detailed CT and MRI findings of 18 patients with pPNETs. (DOCX)

S2 Table Table of chemotherapy (Case no. 8). (DOCX)

S1 Fig Left renal hilum region pPNET in a 36-year-old male (Case no. 8). (DOCX)
