# Supplementary material for: Radiological features of primitive neuroectodermal tumors in intra-abdominal and retroperitoneal regions: A series of 18 cases
Source: PLoS One. 2017 Mar 20;12(3):e0173536. doi: 10.1371/journal.pone.0173536 (PMC5358836; doi:10.1371/journal.pone.0173536)
Supplement: S1 Table — (DOCX) [file pone.0173536.s001.docx]

| **S1 table**: Detailed CT and MRI findings of 18 patients with pPNETs. | | | | | | | | | | | | | | |
| --- | --- | --- | --- | --- | --- | --- | --- | --- | --- | --- | --- | --- | --- | --- |
| Cases no. | Location | Size  (cm) | Margin | Density/intense | Necrosis | Hemorrhage | Cyst | Septa | Calcification | Enhancement | Blood supply arteries/portal of the mass | Tiny feeding arteries | Mild ring enhancement | Other findings |
| 1 | bladder wall | 6.5 | Ill-defined | Moderate, homogenous |  |  |  |  | + | Mild homogenous | — |  |  | — |
| 2 | Right adrenal gland | 5.4 | Well-defined | Moderate, heterogenous | + |  | + | + |  | Intermediate heterogenous | Celiac axis branch/anterior inferior | + | + | Around the renal hilum lymph node metastasis |
| 3 | Presacral area | 6.7 | Ill-defined | Moderate, heterogenous;Isointense (T1WI), heterogeneous mild hyperintense (T2WI) | + |  | + | + |  | Intermediate heterogenous | Left iliac arterial branch/posterior inferior | + |  | mural nodules |
| 4 | Hepatogastric gap | 4.7 | Ill-defined | Moderate, heterogenous | + |  | + | + | + | Intermediate heterogenous | Proper hepatic arterial branch/anterior |  | + | intrahepatic bile duct and main pancreatic duct dilatation |
| 5 | Left adrenal gland | 6.4 | Well-defined | Moderate, heterogenous | + | + |  |  |  | Intermediate heterogenous | Phrenic artery branch/posterior above |  | + | perirenal effusion |
| 6 | Head of the pancreas | 5.6 | Well-defined | Moderate, homogenous |  |  |  |  |  | Mild homogenous | Superior mesenteric arterial branch/posterior |  |  | — |
| 7 | Right adrenal gland | 8.2 | Well-defined | Moderate, heterogenous | + |  | + |  |  | Intermediate heterogenous | Renal arterial branch/bottom left | + | + | Form a tumor draining vein |
| 8 | Left renal hilum | 3 | Ill-defined | Moderate, heterogenous | + |  |  |  |  | Mild heterogenous | Left renal arterial branch/cannot evaluate |  |  | — |
| 9 | Left renal calices | 3.8 | Well-defined | Moderate, homogenous |  |  |  |  | + | Mild homogenous | — |  |  | — |
| **S1 table**: Detailed CT and MRI findings of 18 patients with pPNETs. | | | | | | | | | | | | | | |
| Cases no. | Location | Size  (cm) | Margin | Density/intense | Necrosis | Hemorrhage | Cyst | Septa | Calcification | Enhancement | Blood supply arteries/portal of the mass | Tiny feeding arteries | Mild ring enhancement | Other findings |
| 10 | Left adrenal gland | 3.2 | well-defined | Moderate, hetergenous | + |  |  |  | + | Intermediate hetergenous | Celiac axis branch/anterior |  |  | Retroperitoneal lymph node metastasis |
| 11 | Lesser curvature of stomach beside | 10 | Well-defined | Moderate, heterogenous | + |  |  |  | + | Mild heterogenous | — |  |  | — |
| 12 | Ascending colon | 4 | Ill-defined | Moderate, heterogenous | + |  |  |  |  | Intermediate heterogenous | Superior mesenteric artery branch/ cannot evaluate |  |  | Liver and retroperitoneal lymph node metastasis |
| 13 | Left adrenal gland | 8.8 | Ill-defined | Moderate, heterogenous | + |  | + | + |  | Intermediate heterogenous | Celiac axis branch/ bottom anterior |  |  | — |
| 14 | Mesentery | 10.8 | Well-defined | Moderate, heterogenous | + |  | + |  |  | Intermediate heterogenous | Inferior mesenteric artery branch/posterior | + |  | Retroperitoneal lymph node metastasis |
| 15 | Right renal | 4.4 | Well-defined | Moderate, hetergenous | + |  |  |  |  | Mild hetergenous | Right renal arterial branch/left |  |  | — |
| 16 | Left renal | 10.2 | Ill-defined | Moderate, heterogenous | + |  | + |  | + | Intermediate heterogenous | Left renal arterial branch/ above right | + |  | Left renal vein tumor emboli |
| 17 | Left renal | 12.1 | Ill-defined | Moderate, heterogenous | + | + |  |  |  | Intermediate heterogenous | Left renal arterial branch/ right | + |  | Left renal vein tumor emboli |
| 18 | Hepatogastric gap | 15.9 | Ill-defined | Moderate, hetergenous | + |  | + | + |  | Intermediate hetergenous | Celiac axis branch/anterior | + | + | Invasion into the right pleural |
