# Supplementary material for: Radiological features of primitive neuroectodermal tumors in intra-abdominal and retroperitoneal regions: A series of 18 cases
Source: PLoS One. 2017 Mar 20;12(3):e0173536. doi: 10.1371/journal.pone.0173536 (PMC5358836; doi:10.1371/journal.pone.0173536)
Supplement: S2 Table — (DOCX) [file pone.0173536.s002.docx]

| **S2 table:** Table of chemotherapy (Case no. 8). | |
| --- | --- |
| Date | Project |
| xxxx-xx-xx/ xxxx-xx-xx / xxxx-xx-xx | VAC:VCR2mgd1+EPI80mgd1-2+CTX1.2gd1-2 |
| xxxx-xx-xx/ xxxx-xx-xx / xxxx-xx-xx | IE:IFO3.4gdl1-5+VP-160.19gd1-5 |
| xxxx-xx-xx | VAC:VCR2mgd1+EPI80mgd1-2+CTX1.2gd1-2 |
| xxxx-xx-xx / xxxx-xx-xx | GD:Gemcitabine 1.3gd1+Docetaxel 140mgd1 |
| xxxx-xx-xx / xxxx-xx-xx / xxxx-xx-xx / xxxx-xx-xx xxxx-xx-xx | VIDE:Etoposide 0.2gd1-3+Ifosfamide 3.5g d1-3+Vincristine 2mg d1+Epirubicin 70mg d1-2 |
| xxxx-xx-xx | VDE+Actinomycin D:Ifosfamide 3.5g d1-3+Vincristine 2mg d1+Actinomycin 0.5mg d1-4 |
| xxxx-xx-xx / xxxx-xx-xx | VIT:Irinotecan 40mg d1-5+Vincristine 2mg d1+Temozolomide 200mgd1-5 |
| xxxx-xx-xx / xxxx-xx-xx / xxxx-xx-xx / xxxx-xx-xx / xxxx-xx-xx | VAC+VP-16:VCR2mgd1+EPI80mgd1+CTX1.2gd1-2+Etoposide 0.1g d1-5 |

Note：VCR: Vincristine; EPI: Epirubicin; CTX: Cyclophosphamide; IFO: Ifosfamide; VP-16: Etoposide.
