# Supplementary material for: Radiological features of primitive neuroectodermal tumors in intra-abdominal and retroperitoneal regions: A series of 18 cases
Source: PLoS One. 2017 Mar 20;12(3):e0173536. doi: 10.1371/journal.pone.0173536 (PMC5358836; doi:10.1371/journal.pone.0173536)
Supplement: S1 Fig — (DOCX) [file pone.0173536.s003.docx]

**S1 Fig. Left renal hilum region pPNET in a 36-year-old male (Case no. 8).** The first time contrast-enhanced CT images showed an irregular moderate density lesion in the left renal hilum, which wrapped around the left renal artery and vein (diameter 51 mm) (A). Follow-up CT images obtained 3, 11, 13, 14, 20, 21 and 23 months after chemotherapy (diameter, 43 mm, 82 mm, 80 mm, 92 mm, 93 mm, 98 mm and 105 mm, respectively) (B-H). Response Evaluation Criteria in Solid Tumors evaluation results for SD (stable disease), PD (progressive disease), PD, SD, SD, PD and PD, respectively. The CT images showed that the lesion volume gradually increased, with intermediate heterogeneous enhancement. A large cystic necrosis area was detected. The mass invaded into adjacent tissues, including the left renal artery and vein, left renal parenchyma, abdominal aorta and left psoas major.
